# The modulator role of Urtica dioica on deleterious effects of retinoic acid high doses on histological parameters and fertilization of rats

**DOI:** 10.1016/j.heliyon.2023.e17277

**Published:** 2023-06-14

**Authors:** Shuli Du, Lijuan Wang, Yinghui Wang, Yanna Jin, Aijing Wang, Cuiting Lv, Razzagh Abedi-Firouzjah

**Affiliations:** a Department of Obstetrics, Laoling People’s Hospital, Dezhou, 253600, China; b Department of Gynaecology, Laoling People’s Hospital, Dezhou, 253600, China; c Department of Reproductive Medicine, The Fourth Hospital of Hebei Medical University (Hebei Tuor Hospital), Shijiazhuang, 050000, China; d Department of Medical Physics Radiobiology and Radiation Protection, School of Medicine, Babol University of Medical Sciences, Babol, Iran

**Keywords:** Urtica dioica, Fertilization, Biochemical parameters, Histological parameters, Rats

## Abstract

**Aim:**

This study purposed to evaluate the modulator and protective role of Urtica dioica (UD) extract against deleterious effects of retinoic acid (RA) high doses on histological parameters and fertilization of rats.

**Materials and methods:**

For the in-vivo phase, 60 female Wistar rats were divided into 6 identical groups as 1) control, 2) 25 mg/kg RA, 3) 25 mg/kg UD extract, 4) 50 mg/kg UD extract, 5) UD extract (25 mg/kg) + RA (25 mg/kg), and 6) UD extract (50 mg/kg) + RA (25 mg/kg). Biochemical parameters, including luteinizing hormone (LH), folliclestimulating hormone (FSH), malondialdehyde (MDA) levels, superoxide dismutase (SOD), and catalase (CAT) activities, were measured. In the in-vitro phase, oocytes were obtained from 10 female rats without injection. In addition to the mentioned parameters, histological parameters (oocytes in various stages) and the results of IVM, IVF, and embryo developments were assessed and compared among the groups with the use of one-way ANOVA and Tukey's post hoc tests.

**Results:**

The high dosage of RA significantly reduced the LH and FSH levels; however, UD alone and with RA increased the hormone levels in rats. Regarding the reactive oxygen species (ROS) activity levels in rats' blood samples, RA increased the MDA and decreased the SOD and CAT levels. Treatment with UD extract (UD + RA groups) significantly improved the parameters mentioned, showing UD's antioxidant effect. The rate of oocyte maturation, 2-cell–4-cell and 4-cell–8-cell embryos, and blastocyst formation increased significantly in the groups in which UD extracts were administered compared to the control and RA groups. Furthermore, the increases were significant in the UD + RA groups compared to the RA group.

**Conclusion:**

UD extract can significantly reduce RA high doses side effects on histological parameters and fertilization of rats and has the protective potential against RA deleterious effects.

## 1 Introduction

Since the ovary is an active organ and produces oxidants at high levels, reactive oxygen species (ROS) would block meiosis in oocytes, which prevents embryonic development and induce cell death [1,2]. Therefore, using a culture medium or agent that helps the development of follicles can be the appropriate choice [3].

Urtica dioica (UD), known as nettle, grows in numerous countries and has wide applications in traditional medicine [4]. UD is rich in chemical components (calcium, silicon, iron, and copper), vitamins (A, B, C, E, and K), and bioactive compounds (flavonoids and phenolic acids) [5]. This plant can be useful to treat menstrual and nasal hemorrhage, anemia, pain, menorrhagia, and hair loss [5]. In addition, the studies have determined that UD extracts have some pharmacological characteristics, including antioxidant, antifungal, antiulcer, anticancer, antibacterial, and anti-inflammatory properties [6]. There are many clinical trials and human studies that evaluated the UD effects against several diseases and conditions [[7], [8], [9], [10]]. These studies mostly evaluated the antidiabetic, anti-cancer, and anti-inflammatory potential in patients.

In living systems, oxidants such as ROS and reactive nitrogen species (RNS) may lead to damage macromolecules like proteins, DNA, and lipids which can cause several diseases such as arteriosclerosis, cancer, diabetes, neurodegenerative diseases, and arthritis [11]. Antioxidant activities of UD species were found to be effective in reducing oxidant levels in in-vivo and in-vitro conditions [1,12].

Retinoic acid (RA) is a small lipophilic molecule that is a natural metabolite of vitamin A [13]. RA helps regulate various physiological processes, and its active form has been recognized to support female reproduction [14]. It has been reported that RA in low dosages has dual effects in maturity and embryonic evolution; however, in very high dosages (commonly above 5 mg/kg), it can lead to morphological, biological, and genetic side effects in the cells and tissues, especially in the reproductive system [[14], [15], [16]].

In-vitro maturation (IVM), a modern technology of fertility treatment and preservation [17,18], can increase the chance of fertility in in-vitro conditions. The present study aimed to evaluate the modulator and protective role of UD extract against the deleterious effects of RA high doses on oocyte maturation in rats. We have assessed several biochemical parameters such as luteinizing hormone (LH), folliclestimulating hormone (FSH), malondialdehyde (MDA) levels, superoxide dismutase (SOD), and catalase (CAT) activities, as well as ovary histopathological parameters such as the thicknesses of the theca interna and granulosa layer in antral follicles. Based on our search, although several studies assessed the antioxidant effects of UD extracts, the protective effect of UD on the rats’ fertilization characteristics against the toxic effects of RA high doses was not evaluated in previous studies.

## 2 Materials and methods

All procedures were carried out based on the Animal Experimentation Ethics Committee and National Research Ethics Board (Ethical approval number: MUBABOL.REC.1391.4).

### 2.1 Preparing the UD extract

For preparing hydro-alcoholic UD extract, 100 g of the ground plant was mixed with 200 ml of 50% ethanol. After 72 h, they were centrifuged for 30 min at 1000 g. The mixture was filtered to separate the solution with Whatman filter paper grade no 1. To remove the solvent, the solution was condensed using a rotary vacuum evaporator (INGOS, RVO, 200A) [19]. The obtained extract (weighted 4 gr) was stored in frozen condition at −18 °C until usage. The obtained extracts were diluted in normal saline for injection in mice.

### 2.2 Animals and drug administration

Sixty female Wistar rats were used for the in-vivo phase of this study. Their age range was 6–7 weeks, with a weight of 35 ± 10 gr. The animals were given ad libitum access to food and water, and kept at standard conditions (22 ± 2 °C) and a periodic light/dark.

The female rats were divided into six identical groups: 1) control group: without any intervention, 2) RA group: the animals received 25 mg/kg of RA daily, 3) UD_25_ group: the rats received 25 mg/kg of UD extract daily, 4) UD_50_ group: the rats received 50 mg/kg of UD extract daily, 5) UD_25_ + RA group: 25 mg/kg of UD extract and 25 mg/kg of RA, 6) UD_50_ + RA: 50 mg/kg of UD extract and 25 mg/kg of RA. The doses of UD extract and RA retrieved from previous studies reported the toxic effect of RA [[14], [15], [16]] and the protective effect of UD [[20], [21], [22]]. The UD extract and RA were injected intra-peritoneally (IP) for 7 consecutive days.

In the in-vitro phase of the study, oocytes and sperms were obtained from 10 female and 10 male rats without any injection. The RA and UD agents were also used for IVM (in-vitro maturation) of the oocytes added to immature oocytes culture media [23].

### 2.3 Histopathological parameters

After the last injection day, for histological examinations and in-vivo fertilization (IVF) assessments, the ovaries were removed and fixed in 10% buffered formalin (72 h, room temperature). Ethanol in ascending degrees, Xylene, and paraffin were added to the specimens. Sections with a thickness of 5 μm were stained with hematoxylin (C.I. 75290; Merck) and eosin (C.I. 45380; Sigma). The sections were observed under a light microscope (100X magnification, Zeiss Axioplan, Carl Zeiss, Oberkochen, Germany). The thicknesses of the theca interna and granulosa layer in antral follicles were measured. Based on the ovary morphology, the structures extant in the ovary were categorized into 6 groups; primordial, corpus luteum, primary, preantral, antral, and atretic follicles.

### 2.4 Biochemical parameters

Twenty-four hours after the last injection, the animals were anesthetized by IP injection of 20 mg/kg of Xylazine and 60 mg/kg of Ketamine (Alfasan, Netherlands), and blood samples were collected (1.5 mL) from tail vein. The samples were centrifuged at 3000 rpm for 20 min. LH and FSH in the plasma were quantified by the enzyme-linked immunosorbent assay (ELISA, optical density at 450 nm) based on the manufacturer's guidelines (Zell Bio, Germany, FSH kit catalogue no. RK02819, LH kit catalogue no. RK02986).

For measuring MDA levels in the samples, 2 ml of thiobarbituric acid (TBA) reactant was added to 1 ml of the sample in hot water bath heating [24]. The samples were removed after 40 min and allowed to reach room temperature, and centrifuged for 10 min. The optical density of MDA was determined at 532 nm using a spectrophotometer (UV-1800, Shimadzu, japan) using rat malondialdehyde ELISA kit (MyBioSource, Inc. USA, catalogue no. MBS738685). Activity levels of SOD were measured based on Madesh and Balasubramanian protocol design [25]. This protocol is a colorimetric assay involving the generation of superoxide by pyrogallol auto-oxidation and the inhibition of superoxide-dependent reduction in the tetrazolium dye, measured at 420 nm. Activity levels of CAT activity were obtained according to Aebi method [26], which is principally based on the demonstration of the rate constant of hydrogen peroxide decomposition. The CAT activity was measured at 240 nm using a spectrophotometer (UV-1800, Shimadzu, japan).

### 2.5 Oocyte, sperm collection, and in-vivo fertilization (IVF)

Pregnant mare serum gonadotropin (PMSG) hormone with 5 international units (IU) was injected IP to induce the proliferation and development of oocytes in rats. The female animals (that have not received any injection) were killed by cervical dislocation. After that, the cumulus-oocyte complex (COC, i.e., the oocyte and cumulus cells) was collected from the ampulla region of both sides of the uterus and transferred to Petri dishes containing human fallopian tubes (HFT), and immature germinal vesicle (GV) oocytes were obtained. In total, 750 oocytes were obtained and randomly assigned into 6 identical groups, similar to the in-vivo phase groups, including 1) control group, 2) RA group: the cells were kept in cell culture media with a concentration of 25 μg/ml of RA for 24 h, 3) UD_25_ group: the cells were kept in cell culture media with a concentration of 25 μg/ml of UD for 24 h, 4) UD_50_ group: the cells were kept in cell culture media with a concentration of 50 μg/ml of UD for 24 h, 5) UD_25_ + RA group: the cells were kept in cell culture media with concentrations of 25 μg/ml of RA and UD for 24 h, 6) UD_50_ + RA: the cells were kept in cell culture media with concentrations of 25 μg/ml of RA and 50 μg/ml UD for 24 h.

Several agents, including recombinant human follicular stimulating hormone (Organon, Oss, The Netherlands, 7.5 IU/mL), MEM-α with penicillin (100 IU), fetal bovine serum, streptomycin (100 IU), and human chorionic gonadotrophin (Organon, Oss, The Netherlands, 100 IU/mL) were used to prepare the IVM medium. RA and UD extract were added in single doses to a standard rich culture medium. About 10–15 cumulus-oocyte complexes were transferred to 25 μL drops, which were covered by mineral oil.

Furthermore, sperm were collected from male rats for the IVF procedure. Healthy male animals were killed with cervical dislocation, and their vas deferens and epididymis tails were removed and transferred to Petri dishes containing HFT culture medium. The incubation temperature for the culture medium was 37 °C under 5% CO_2_. After IVM, metaphase II oocytes were washed in IVF media and transferred to (three or four oocytes) 50 μL microdroplets, previously covered by mineral oil. For IVF, about 2 × 10^6^ spermatozoa/mL were added to the droplets comprising oocytes, and incubated for 4–6 h (at 37 °C with 5% CO_2_). The embryos were cleaned in the droplets of potassium simplex optimization medium (KSOM with 4 mg/mL bovine serum albumin) and subsequently transferred to the Petri dish. The numbers of two-cell, morula, four-cell, and blastocyst embryos were recorded during IVC (96 h), using a light microscope (100X magnification, Zeiss Axioplan, Carl Zeiss, Oberkochen, Germany) daily.

### 2.6 Statistical analysis

The normality distribution of the data of the assessed parameters was evaluated by Kolmogorov-Smirnov (K–S) test. The SPSS software package (v. 22, SPSS Inc., Chicago, IL, USA) was used for all of the statistical tests. The data were compared using a one-way analysis of variance (ANOVA) and post hoc Tukey tests. P values of <0.05 were considered statistically significant.

## 3 Results

### 3.1 Biochemical parameters

#### 3.1.1 LH and FSH levels

**Fig. 1:**
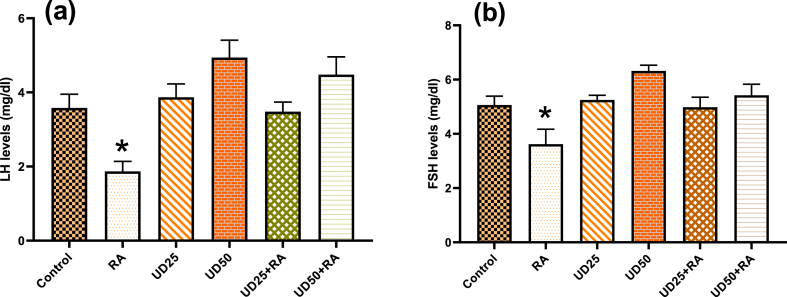
(a): Luteinizing hormone (LH) and (b): folliclestimulating hormone (FSH) levels in the blood serums of rats in the control, RA, UD_25_, UD_50,_ UD_25_ + RA, and UD_50_ + RA groups. *: significantly differs from the other groups (P < 0.05). Note: only some main significant levels are shown in the figures.

LH and FSH levels (mg/dl) are presented for various investigated groups (Fig. 1). According to Fig. 1a, LH levels showed that there were statistically significant differences among the evaluated groups (P = 0.00). Post hoc Tukey statistical tests also showed that animals in the “UD_50_” and “UD_50_ + RA” groups had no significant differences (P = 0.187). Furthermore, the animals in the “UD_25_” and “UD_25_ + RA” groups did not show significant differences with each other (P = 0.367) and control group (P > 0.89), showing the protective effect of UD extract alone or with RA. Other groups had significant differences from each other regarding the LH values (P < 0.014).

Regarding Fig. 1b, there are statistically significant differences in FSH levels among the assessed groups (P = 0.00). Post hoc Tukey tests showed no significant differences in FSH levels between the animals in the “control”, “UD_25_”, “UD_25_ + RA”, and “UD_50_ + RA” groups (P > 0.05). Other groups had significant differences from each other regarding the FSH levels (P < 0.014).

#### 3.1.2 MDA, SOD, and CAT levels

**Fig. 2:**
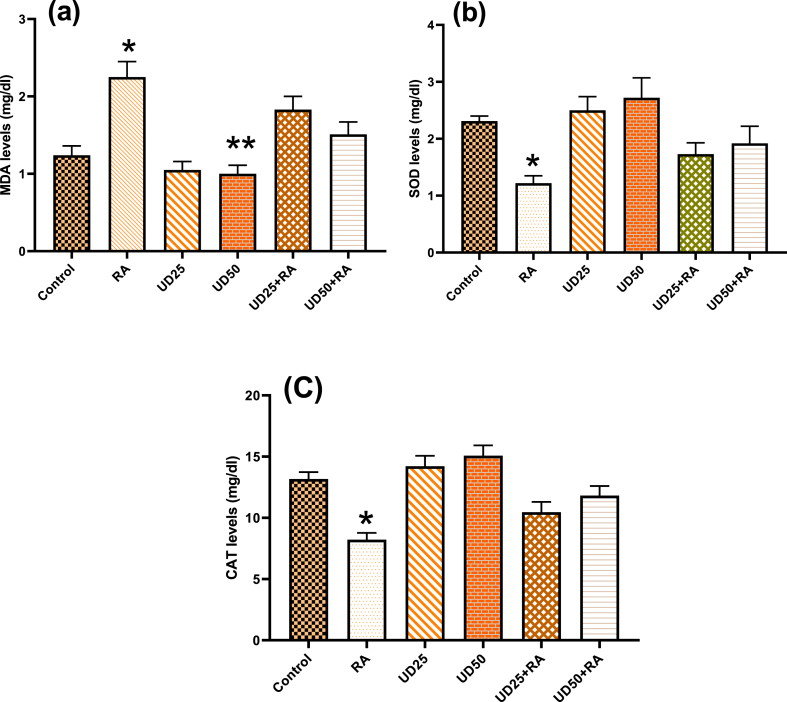
(a): Malondialdehyde (MDA), (b): superoxide dismutase (SOD), (c): catalase (CAT) levels in the blood serums of rats in the control, RA, UD_25_, UD_50,_ UD_25_ + RA, and UD_50_ + RA groups. *: significantly differs from the other groups (P < 0.05). **: significantly differs from the other groups except with UD_25._ Note: only some main significant levels are shown in the figures.

The MDA, SOD, and CAT levels in the blood serums of rats are presented in Fig. 2. A significant increase was observed in the MDA levels (Fig. 2a) of the rats in the RA group (P = 0.000) compared to the control group and rats with UD treatment (P < 0.001). Post hoc Tukey test results showed that treatment with UD extract could significantly reduce the increased levels of MDA in the RA rat (P < 0.005). Additionally, it was not observed any significant differences in the MDA levels between the UD alone (i.e., “UD_50_” and “UD_25_” groups) groups (P = 0.971). However, animals in the “UD_50_” group illustrated a significant difference from the control group (P = 0.066).

Fig. 2b shows the mean and standard deviation values of SOD levels in the rats of the evaluated groups. The one-way ANOVA statistical test showed statistical differences among the groups (P = 0.000). It was found that the SOD levels in the RA group were lower than that of control rats (P = 0.000). Post hoc Tukey tests showed that rats in UD-only treatment groups (i.e., “UD_50_” and “UD_25_” groups) had no significant differences with rats in the control group (P > 0.07). Although UD extracts significantly increased the SOD levels of the samples compared to the RA alone (P < 0.001), the increase in SOD levels of “UD_25_ + RA” and “UD_50_ + RA” were significantly lower compared to the control groups (P < 0.001).

Fig. 2c illustrates the effect of RA and UD extract on the CAT activity among all the groups. The statistical differences in CAT activity were found using a one-way ANOVA statistical test (P = 0.000). The CAT levels had significantly lower values in the RA group compared with other groups (P < 0.001), showing that treatment with UD can significantly ameliorate RA's oxidant effects. It was also seen that the CAT levels were increased significantly in the rats of the “UD_50_” group compared to the control and RA groups (P < 0.008). Furthermore, the CAT levels in the “UD_25_ + RA” and “UD_50_ + RA” groups were significantly lower compared to relevant UD treatment and control groups (P < 0.025).

### 3.2 Histological parameters

**Fig. 3:**
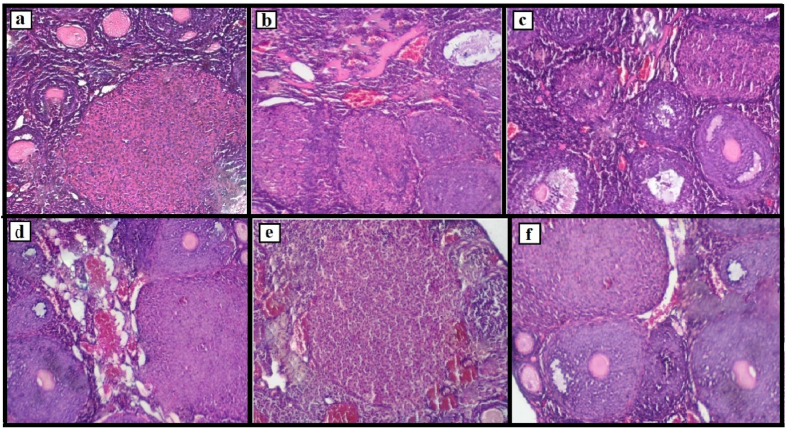
Ovarian histological images (magnification 40X). (a) and (b) are related to the control and UD_50_ groups, respectively (epithelial tissue of the ovary surface to simple cubic forms; various types of follicles, including resting and growing follicles such as antral, preantral, and primary are observed). (c): RA group, showing antral, preantral, and primary follicles which are mostly with atresia. (d): UD_25_ group, most types of follicles (primary, preantral, antral, and corpus luteum) are observed. The number of mature oocytes was increased in this group compared to the control and RA groups. (e) and (f) are related to RA + UD_50_ and RA + UD_25_ groups, respectively, showing various kinds of developing and healthy follicles along with a number of follicles undergoing atresia.

The sample images of the ovarian tissue for the investigated groups are presented in Fig. 3. Fig. 3(a) and (b) are related to the control and UD_50_ groups, respectively; Fig. 3(c) is the RA group; Fig. 3(d) is the UD_25_ group, and Fig. 3(e) and (f) are related to the RA + UD_50_ and RA + UD_25_ groups, respectively. In addition, the number of primordial, primary follicles, preantral follicles, antral follicles, corpus luteum, and atretic follicles in ovary tissues in the assessed groups are depicted in Table 1. Primordial follicle numbers in the assessed groups were measured and no significant difference was found among the groups (P = 0.148). The results of post hoc Tukey statistical test also showed no significant differences between the groups compared with each other (p > 0.23).

**Table 1:** The number of primordial, primary follicles, preantral follicles, antral follicles, corpus luteum, and atretic follicles in ovary tissues in the control, RA, UD_25_, UD_50,_ UD_25_ + RA, and UD_50_ + RA groups.

Parameters	Primordial	Primary	Preantral	Antral	Atretic	Corpus-luteum
Groups						
**Control**	22.12 ± 1.53	5.11 ± 1.31	16.65 ± 1.69	4.41 ± 0.82	4.32 ± 1.35	8.51 ± 1.45
**RA**	23.34 ± 1.48	9.48 ± 1.71	27.32 ± 2.87	5.77 ± 1.25	11.42 ± 2.11	4.28 ± 1.11
**UD**_**25**_	23.18 ± 1.77	6.69 ± 1.61	18.71 ± 2.05	7.36 ± 1.42	3.25 ± 0.96	10.25 ± 1.72
**UD**_**50**_	24.02 ± 1.56	7.72 ± 1.44	21.41 ± 2.21	8.22 ± 1.54	2.22 ± 0.67	12.75 ± 1.54
**UD**_**25**_ + **RA**	22.51 ± 1.60	7.73 ± 1.39	20.44 ± 1.91	6.74 ± 1.39	6.27 ± 1.74	6.22 ± 1.07
**UD**_**50**_ + **RA**	23.35 ± 1.55	8.65 ± 1.52	24.17 ± 2.18	7.11 ± 1.29	5.73 ± 1.59	8.08 ± 1.12
**P-value**	0.148	0.007	0.000	0.000	0.000	0.000

The results of the one-way ANOVA test showed a significant difference among the groups for the number of primary and preantral follicles (P < 0.008). Significant increases in the primary and preantral follicles were observed after the delivery of 25 mg/kg RA using IP injection in the rats for seven consecutive days (RA group) in comparison with the control group (P < 0.05). Furthermore, injection of UD extracts in both assessed concentrations increased the number of primary and preantral follicles compared to the control group, in which this increase was not significant (P = 0.085) in the “UD_25_” group. However, this increase was significant in “UD_50_”, “UD_25_ + RA”, and “UD_50_ + RA” compared to the control group (P < 0.01), and they had no significant differences with each other (P > 0.15).

The number of antral follicles in the assessed rats showed significant differences among the groups (P = 0.000). The results of the post hoc Tukey statistical test showed significant differences between the UD groups (UD_25_, UD_50_, UD_25_ + RA, and UD_50_ + RA) and control group (p < 0.009). Although RA and/or UD injections significantly increased the number of antral follicles compared to the control group, this increase was remarkably higher for rats in the “UD_25_” and “UD_50_” groups.

Atretic follicles numbers in the assessed groups had significant differences (P = 0.000). Administration of RA significantly increased this parameter compared to the control group (P < 0.001). Furthermore, IP injection of UD extract decreased the atretic follicle numbers with a significant change in “UD_50_” (P = 0.033) and no significant changes in “UD_25_” (P = 0.115) groups compared to the control group. Although the combination of RA and UD extract (in both concentrations) decreased the number of atretic follicles in the “UD_25_ + RA” and “UD_50_ + RA” groups, these changes were not significant in comparison with the control group (P > 0.101).

We also found a significant difference among the groups regarding the number of corpus-luteum follicles (P = 0.000). Post-hoc Tukey tests showed that the rats in the RA group had significantly lower corpus-luteum follicle numbers (P = 0.004), and the UD_50_ group had significantly higher numbers compared to the control group (P = 0.021). In other groups, there were no significant differences between the control group and each other (P > 0.108).

### 3.3 IVM, IVF, and early embryo development

**Fig. 4:**
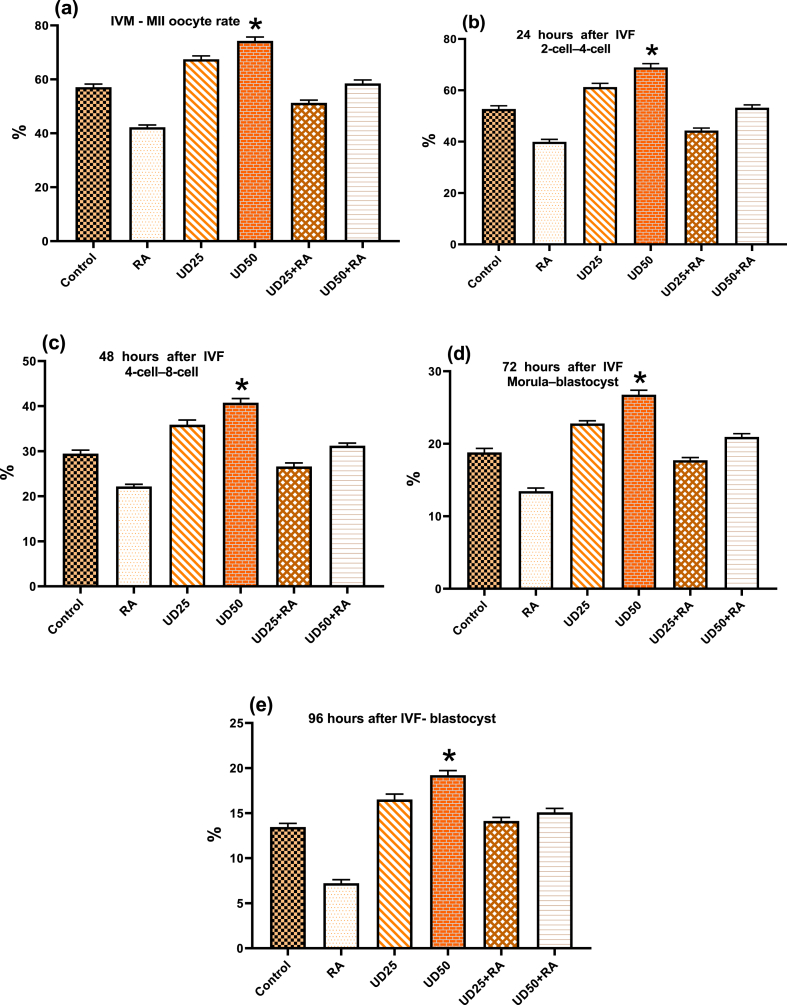
Percentage of *in vitro* maturation (IVM) - MII oocyte rate (a), 24 h after *in vitro* fertilization (IVF) 2-cell–4-cell (b), 48 h after IVF 4-cell–8-cell (c), 72 h after IVF Morula–blastocyst (d), and 96 h after IVF- blastocyst (e) in the control, RA, UD_25_, UD_50,_ UD_25_ + RA, and UD_50_ + RA groups. *: significantly differs from the other groups except with UD_25_. Note: Only some main significant levels are shown in the figures.

Fig. 4 shows (a) the percentage of IVM - MII oocytes, (b) IVF 2-cell–4-cell embryos after 24 h, (c) IVF 4-cell–8-cell embryos after 48 h, (d) IVF Morula–blastocysts after 72 h, and (e) IVF- blastocysts after 96 h for the studied groups. As shown in this figure, the rate of oocyte maturation, 2-cell–4-cell and 4-cell–8-cell embryos, and blastocyst formation increased significantly in the groups in which UD extracts were administered (UD_25_ and UD_50_ groups) compared to the control and RA groups (P < 0.001). Furthermore, these increases were significant in the groups that received UD extract after RA administration (UD_25_ + RA and UD_50_ + RA groups) compared to the RA group (P = 0.000). This demonstrates the adverse effects of RA high doses and the ameliorative effect of UD extract on the embryo's survival.

## 4 Discussion

In this experimental work, we have assessed the ameliorative role of UD extract on oocyte maturation against deleterious effects of RA (in a high dosage, which causes side effects in oocyte cells). Positive effects of UD extract have been reported in several studies for controlling pro-inflammatory cytokine, immunologic response to stress, glucose transporters, and lipid peroxidation in different organs [[27], [28], [29]]. For example, Yıldızhan et al. [30] investigated the UD protective effects on liver tissue injury and antioxidant capacity in irradiated rats, and revealed that UD extract reduces the radiation oxidative stress and degenerative changes of liver.

Many studies reported the protective effect of natural agents or herbal extracts on fertilization parameters or sperm health [[31], [32], [33], [34]] or other side effects/toxicities [[35], [36], [37], [38]]. For instance, Raeeszadeh et al. [31] compared the effects of broccoli extract with vitamins C and E on oxidative damage, sperm quality parameters, and reproductive hormones in rats. They reported a significantly higher protective effect of broccoli extracts for rat sperm against oxidative damage during cryopreservation and improved reproductive performance. In another study [31], the effects of N-acetylcysteine and vitamin E were evaluated on cyclophosphamide-induced ovarian damage. It was reported that N-acetylcysteine and vitamin E co-administration could significantly decrease the side effects of cyclophosphamide, especially in ovarian tissue. In addition, other studies approved the UD protective effect on the reproductive system [39,40]. For example, Jalili et al. [40] assessed the protective effect of UD extract against nicotine-induced damage on sperm cell viability, motility, count, testis histology, and testosterone hormone in mice. They revealed that UD, which has anti-oxidative and anti-inflammatory properties, could inhibit nicotine’s adverse effects on the abovementioned parameters.

We assessed LH and FSH hormone levels in the rats’ peripheral blood samples to illustrate the effects of RA and UD extracts. LH and FSH play vital roles in gonadal function and are frequently used in assisted reproductive technology. LH stimulates follicular growth and ovulation in synergy with FSH [41]. According to our results, IP injection of RA high doses decreased the LH and FSH levels significantly compared to the control group. However, injection of UD extract along with the RA can inhibit the decrease in FSH and LH levels in rats. Similar to our findings, several studies reported the ameliorative effects of natural plant extracts on sexual hormones [[42], [43], [44]]. For example, Chauhan et al. [42] approved the protective effects of Pueraria tuberosa DC extract in LH and FSH levels and androgenesis in rats.

MDA, CAT, and SOD are the enzymes that defend against free radical complications, and their levels can be considered as reference values of oxidative stress parameters [45]. In the present study, RA high doses resulted in more significant free radicals and higher MDA levels as the ROS-induced lipid peroxidation end product. In line with our study, Telo et al. [46] assessed the effects of UD on antioxidant enzyme activities and mammary gland cancer in rat models. Based on their results, there was a significant increase in plasma MDA levels of N-methyl-N-nitrosourea (NMU) group (given 50 mg/kg NMU by IP injection) compared with an untreated group and a UD group (fed with 50 g/kg UD). In addition, the SOD activity of the erythrocytes was significantly decreased in the NMU group treated with the UD compared to other groups. The CAT activity was significantly increased in the UD group compared with the NMU group and NMU + UD group. Their findings demonstrated that the UD components might have effects on some antioxidant enzyme activities and lipid peroxidation. In another study, Aktas et al. [47] examined the effects of UD in the testicular I/R model. They have found a decrease in the MDA levels and an increase in the activities of SOD, CAT, and glutathione peroxidase in I/R + UD groups compared to I/R group. They concluded that the protective effects of the UD extract are via reduction of histological damage, apoptosis, oxidative stress, and lipid peroxidation. Similar to previous studies, we also found that UD extract can increase the CAT and SOD levels significantly in comparison with the control group and meliorate the RA damages if it was delivered along with RA to the rats.

Higher levels of ROS adversely affect IVF procedures, embryo development, and clinical pregnancy rates. Therefore, obtaining good-quality oocytes before IVF is one of the essential steps in this procedure [48]. In this regard, we showed that application of UD extract, as a natural antioxidant, prior to the IVF procedure can reduce the ROS levels and provide high-quality oocytes.

Several studies describe the positive effects of RA and retinol metabolites on IVM and embryo development [49,50]. RA and its metabolites are important factors in the embryos’ formation. It was reported that retinol could improve the viability of embryos at *in vitro* conditions [49]. It has also been shown that RA can improve the development of the oocyte during the IVM procedure [51]. We also found that RA, even in high doses, can increase the number of primary, antral, atretic and preantral follicles; however, it led to a decrease in corpus-luteum follicle numbers. Vitamins and flavonoids of the UD extracts prevent the destruction of oocytes and reduce oocyte death [52]. Kanter et al. [53] also reported that the effects of CCL4 administration, which causes an increase in the peroxidation of lipids and hepatic enzymes, as well as a reduction in the antioxidant levels, can be moderated by UD extracts.

We have found a higher maturation rate and early embryonic development in the presence of UD extract, demonstrating the positive effects of UD extract during *in vitro* culture. Notably, this effect was dose-dependent, in which a higher UD dosage increased the percentage of oocyte maturation and embryonic development. In a way that, the groups with higher UD extracts (UD_50_ and UD_50_ + RA groups) had higher *in vitro* developments, including IVM, IVF, and IVC rates of oocytes. Similar to our findings, several studies demonstrated the improvement effect of plant extracts with antioxidant properties on *in vitro* development and IVM of animal oocytes [48,54,55]. The positive effects of UD extract can be related to oocyte cytoplasmic maturation promotion through direct or indirect modulatory effects on the gene expression pattern of gonadotropin receptors. UD contains phenolic compounds (especially flavonoids) that generally have antioxidant potential and protect cells against oxidative stress.

The limitations of our study could be referred to considering two concentrations of UD (25 and 50 mg/kg) and one concentration of RA (25 mg/kg); higher or lower values of UD concentrations can be evaluated for future studies to find the optimum concentration. In addition, the protective effect of UD can be compared with other natural antioxidant agents like Chinese yam polysaccharide. From the clinical perspective, the UD extract can be considered a potential protector/modulator for fertilization function which can be investigated for future clinical trial studies.

## 5 Conclusion

The present study supported that UD extract can significantly reduce RA high doses side effects on histological parameters and fertilization of rats, and has the healing potential against the RA deleterious effects. Further studies are needed to clarify the exact mechanism of UD extract.

## Ethical consideration

All procedures were carried out based on the Animal Experimentation Ethics Committee and National Research Ethics Board (Babol University of Medical Sciences; ethical approval number: MUBABOL.REC.1391.4).

## Data availability

The data used to support the findings of this study are available from the corresponding author upon request.

## Author contribution statement

Shuli Du, Lijuan Wang, Yinghui Wang: Conceived and designed the experiments; Performed the experiments; Analyzed and interpreted the data. Yanna Jin, Aijing Wang, Cuiting Lv, Razzagh Abedi-Firouzjah: Contributed reagents, materials, analysis tools or data; Wrote the paper.

## Declaration of competing interest

The authors declare that they have no known competing financial interests or personal relationships that could have appeared to influence the work reported in this paper.
